# Resistance profile of clinically relevant bacterial isolates against fluoroquinolone in Ethiopia: a systematic review and meta-analysis

**DOI:** 10.1186/s40360-018-0274-6

**Published:** 2018-12-12

**Authors:** Mekonnen Sisay, Fitsum Weldegebreal, Tewodros Tesfa, Zerihun Ataro, Dadi Marami, Habtamu Mitiku, Birhanu Motbaynor, Zelalem Teklemariam

**Affiliations:** 1 Department of Pharmacology and Toxicology, School of Pharmacy, College of Health and Medical Sciences, Haramaya University, P.O.Box 235, Harar, Ethiopia; 2 Department of Medical Laboratory Sciences, College of Health and Medical Sciences, Haramaya University, P.O.Box 235, Harar, Ethiopia; 3 Department of Pharmaceutical Analysis, School of Pharmacy, College of Health and Medical Sciences, Haramaya University, P.O. Box 235, Harar, Ethiopia

**Keywords:** Bacterial isolates, Resistance, Fluoroquinolone, Ciprofloxacin, Ethiopia

## Abstract

**Background:**

Fluoroquinolones are among the most frequently utilized antibacterial agents in developing countries like Ethiopia. Ciprofloxacin has become the most prescribed drug within this class and remains as one of the top three antibacterial agents prescribed in Ethiopia. However, several studies indicated that there is a gradual increase of antibacterial resistance. Therefore, this meta-analysis aimed to quantitatively estimate the prevalence of ciprofloxacin resistance bacterial isolates in Ethiopia.

**Methods:**

Literature search was conducted from electronic databases and indexing services including EMBASE (Ovid interface), PubMed/MEDLINE, Google Scholar, Science Direct and WorldCat. Data were extracted with structured format prepared in Microsoft Excel and exported to STATA 15.0 software for the analyses. Pooled estimation of outcomes was performed with DerSimonian-Laird random-effects model at 95% confidence level. Degree of heterogeneity of studies was presented with I^2^ statistics. Publication bias was conducted with comprehensive meta-analysis version 3 software and presented with funnel plots of standard error supplemented by Begg’s and Egger’s tests. The study protocol has been registered on PROSPERO with reference number ID: CRD42018097047.

**Results:**

A total of 37 studies were included for this study. The pooled prevalence of resistance in selected gram-positive bacterial isolates against ciprofloxacin was found to be 19.0% (95% confidence interval [CI]: 15.0, 23.0). The degree of resistance among *Staphylococcus aureus*, Coagulase negative Staphyloccoci (CoNS), *Enterococcus faecalis* and Group B Streptococci (GBS) was found to be 18.6, 21.6, 23.9, and 7.40%, respectively. The pooled prevalence of resistance in gram-negative bacteria was about 21.0% (95% CI: 17, 25). Higher estimates were observed in *Neisseria gonorrhea* (48.1%), *Escherichia coli* (24.3%) and *Klebsiella pneumonia* (23.2%). Subgroup analysis indicated that blood and urine were found to be a major source of resistant *S. aureus* isolates. Urine was also a major source of resistant strains for CoNS, Klebsiella and Proteus species.

**Conclusion:**

Among gram-positive bacteria, high prevalence of resistance was observed in *E. faecalis* and CoNS whereas relatively low estimate of resistance was observed among GBS isolates. Within gram-negative bacteria, nearly half of isolates in *N. gonorrhoea* were found ciprofloxacin resistant. From enterobacteriaceae isolates, *K. pneumonia* and *E. coli* showed higher estimates of ciprofloxacin resistance.

**Electronic supplementary material:**

The online version of this article (10.1186/s40360-018-0274-6) contains supplementary material, which is available to authorized users.

## Background

Quinolones are groups of antibacterial drugs having an extensive application in both clinical and veterinary medicine. The older (first generation) quinolones including nalidixic acid and cinoxacin were primarily used for the treatment of urinary tract infections as their concentration in urine is relatively higher than that of the plasma. In 1980s, the introduction of fluorinated derivatives (fluoroquinolones) such as ciprofloxacin and norfloxacin became a major breakthrough in the development of relatively safer, orally effective and entirely synthetic broad spectrum antibacterial agents [1, 2]. As a result, quinolones have been routinely used for several bacterial infections. Recently, ciprofloxacin was pointed out as the most consumed antibacterial agent world-wide. Within a second generation quinolones, it has a sound medical importance in treating infections caused by many enterobacteriaceae and other gram-negative bacilli. Ciprofloxacin is the most potent of fluoroquinolones for pseudomonal infections associated with cystic fibrosis. However, their widespread use with some degree of evidence of misuse or use of these agents to micro-organisms to which they have poor activity has been blamed for the rapid development of resistance to these agents [3, 4].

In Ethiopia, ciprofloxacin has become the most commonly utilized fluoroquinolone and one of the top three antibacterial agents in clinical practice [5–8]. Study conducted by Birru et al. indicated that there is a high degree of inappropriate use of ciprofloxacin. The study emphasized that nearly half of the treatment was shown to have inappropriate dosage regimen with the duration of therapy being the dominant one in Boru Meda Hospital [9]. Such inappropriate use paves a way forward for the emergence and spread of antimicrobial resistance (AMR). AMR can result from mutations in housekeeping structural or regulatory genes as well as from horizontal acquisition of foreign genetic information [10–12]. Resistance to the quinolones often emerges at low-levels by acquisition of an initial resistance conferring mutation. Acquisition of subsequent mutations leads to higher levels of resistance against second and newer-generation quinolones such as ciprofloxacin [13]. At present, AMR is resulting in increased morbidity, mortality, and healthcare costs in developing countries [14]. This study is, therefore, aimed to quantitatively estimate ciprofloxacin resistance among clinically relevant bacterial isolates in Ethiopia.

## Methods

### Study protocol

The identification of records, screening of titles and abstracts as well as evaluation of eligibility of full texts for final inclusion was conducted in accordance with the Preferred Reporting Items for Systematic review and Meta-analysis (PRISMA) flow diagram [15]. PRISMA checklist [16] was also strictly followed while conducting this systematic review and meta-analysis. The completed checklist has been provided as supplementary material (Additional file 1: Table S1). The study protocol is registered on PROSPERO with reference number ID: CRD42018097047 and the published methodology is available online from: http://www.crd.york.ac.uk/PROSPERO/display_record.php?ID=CRD42018097047

### Identification of records and search strategy

Literature search was carried out through visiting legitimate databases and indexing services-PubMed/MEDLINE, EMBASE (Ovid interface) and other supplementary sources including Google Scholar, WorldCat catalog, ResearchGate and Cochrane library. Advanced search strategies were applied in major databases to retrieve relevant findings closely related to resistance/susceptibility of isolates to ciprofloxacin. Articles published in subscription based journals under Science-Direct and Wiley online library were accessed through HINARI:WHO for developing countries. The search was conducted with the aid of carefully selected key-words and indexing terms within specified time (online records from 2015- May, 2018). Excluding the non-explanatory terms, the search strategy included “quinolone [MeSH]”, ciprofloxacin [MeSH], “antimicrobial susceptibility”, “antimicrobial resistance”, “antibacterial sensitivity” and “Ethiopia”. Boolean operators (AND, OR), truncation and MeSH terms were used appropriately for systematic identification of records for the research question. The search was conducted from 25 April to 10 May, 2018 and all published and unpublished articles available online till the day of data collection were considered. Gray literatures from organizations and online university repositories were accessed through Google Scholar and WorldCat.

### Screening and eligibility of studies

Records identified from various electronic databases, indexing services and directories were exported to ENDNOTE reference software version 8.2 (Thomson Reuters, Stamford, CT, USA) with compatible formats. Duplicate records were identified, recorded and removed with ENDNOTE. Some duplicates were addressed manually due to variation in reference styles across sources. Thereafter, two authors (MS and FW) independently screened the title and abstracts with predefined inclusion criteria. Two authors (MS and TT) also independently collected full texts and evaluated the eligibility of them for final inclusion. In each case, the rest authors played a critical role in solving discrepancies arose between two authors to come into consensus.

### Inclusion and exclusion criteria

During initial screening of titles and abstracts as well as evaluating full texts for eligibility, there have been predefined inclusion-exclusion criteria. Cross sectional studies addressing the prevalence of ciprofloxacin-resistant bacterial isolates obtained from human source (patients) regardless of the clinical characteristics and nature of specimen were included. Only English language literatures and online records published from 2015 to May, 2018 were considered for further eligibility assessment. All review articles and original articles conducted outside of Ethiopia were excluded during initial screening. Articles with irretrievable full texts (after requesting full texts from the corresponding authors via email and/or ResearchGate), records with unrelated outcome measures, articles with missing or insufficient outcomes were excluded.

### Data extraction

With the help of standardized data abstraction format prepared in Microsoft Excel (Additional file 2: Table S2), two authors (MS and HM) independently extracted important data related to study characteristics (study area, first author, year of publication, study design, patient characteristics, source of isolates, types of isolates, and number of isolates) and outcome of interest (number of resistant isolates for each bacterium).

### Critical appraisal of studies

The quality of studies was evaluated according to Newcastle-Ottawa scale adapted for cross-sectional studies [17] and graded out of 10 points (stars). For ease of assessment, the tool has included important indicators categorized in to three major sections: 1) the first secstion assesses the methodological quality of each study and weighs a maximum of five stars 2) the second section considers comparability of the study and takes 2 stars 3) the remaining section assess outcomes with related to statistical analysis. This critical appraisal was conducted to assess the internal (systematic error) and external validity of studies and to reduce the risk of biases in individual studies. The mean score of two authors were taken for final decision and studies with score greater than or equal to five were included.

### Outcome measurements

The primary outcome measure is the prevalence of ciprofloxacin resistant bacterial isolates in Ethiopia. It is aimed to assess the pooled estimates of antibacterial resistance at the national level. The measurement was conducted for selected gram-positive (*Staphylococcous aureus*; Coagulase negative staphylococci (CoNS), Group B Streptococci (GBS) and *Enterococcus faecalis)* and gram-negative bacterial isolates (*Escherichia coli*, *Klebsiella pneumonia*, *Pseudomonas aueroginosa*, Proteus species, *Neisseria gonorrhea*, and other enteric microorganisms) obtained from patients with presumed or confirmed infectious diseases. Subgroup analysis was also conducted based on the source of bacterial isolates.

### Data processing and statistical analysis

The relevant data were extracted from included studies using format prepared in Microsoft Excel and exported to STATA 15.0 for outcome measures and subgroup analyses. Considering variation in true effect sizes across population (clinical heterogeneity), Der Simonian and Laird’s random effects model was applied for the analyses at 95% confidence level. Heterogeneity of studies was determined using I^2^ statistics. Comprehensive Meta-analysis version-3 software (Biostat, Englewood, New Jersey, USA) was used for publication bias assessment. For gram-positive and gram-negative bacterial isolates, the presence of publication bias was evaluated by using the Begg’s and Egger’s tests and presented with funnel plots of standard error of Logit event rate [18, 19]. A statistical test with a *p*-value less than 0.05 (one tailed) was considered significant.

## Results

### Search results

**Fig. 1:**
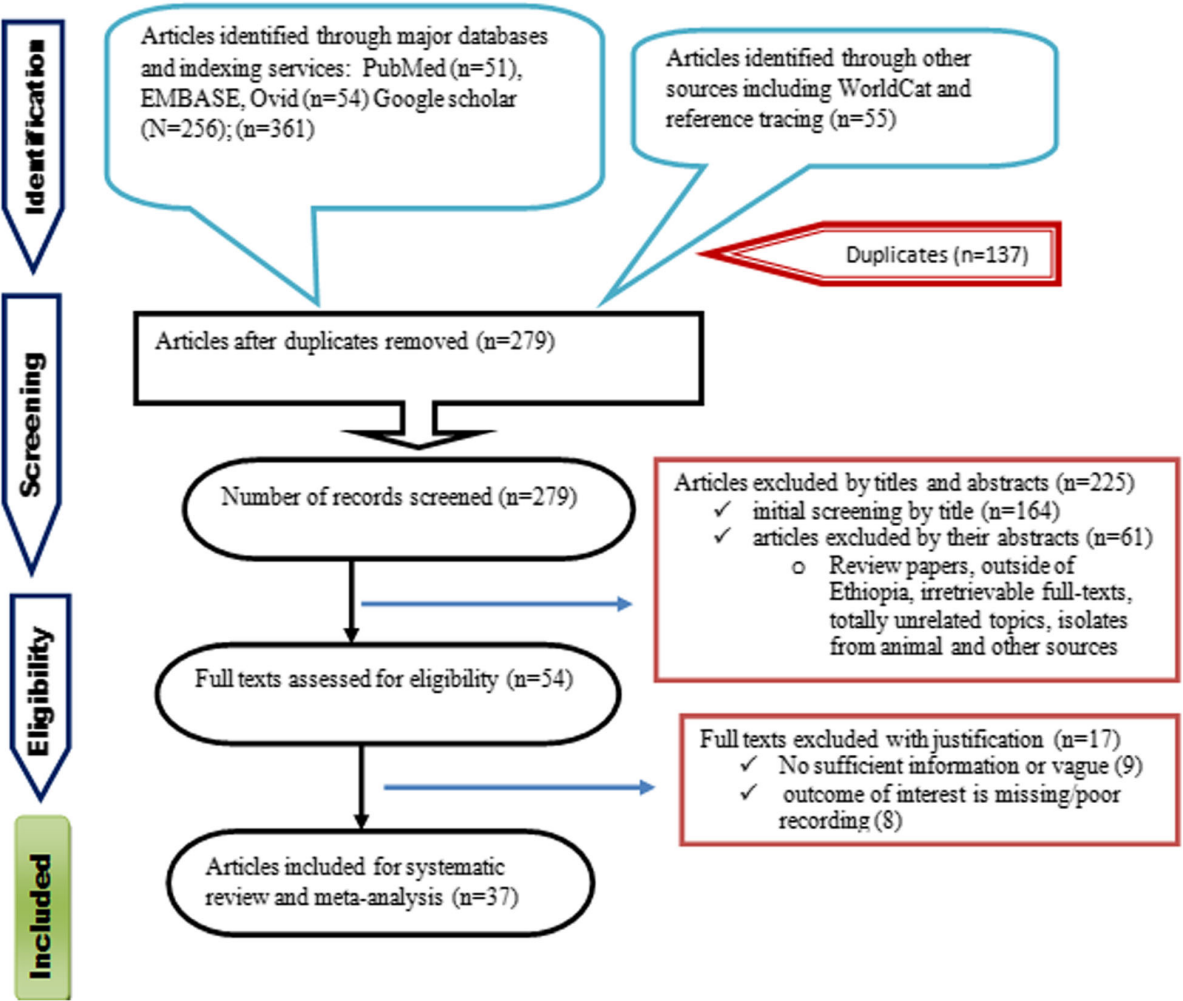
PRISMA flow chart describing the selection process

A total of 416 records were identified from several sources including PubMed/MEDLINE, EMBASE, Google Scholar, Science Direct and WorldCat catalog. From these, 137 duplicate articles were removed with the help of ENDNOTE and manual tracing. The remaining 279 records were screened using their titles and abstracts and 225 of them were excluded. Full texts of 54 records were then evaluated for eligibility. From these, 17 articles were also excluded as the outcome of interest was found missing, insufficient and/or ambiguous. Finally, 37 articles have passed the eligibility criteria and quality assessment and hence included in the study (Fig. 1).

### Study characteristics

As shown in Table 1, a total of 37 studies with 3235 selected bacterial isolates (1303 gram-positive and 1932 gram-negative) were included for systematic review and meta-analysis. We included studies that employed both retrospective and prospective cross-sectional study design. The year of publication of included studies ranged from 2015 to 10 May 2018 since antimicrobial resistance is highly time-sensitive. The study included a wide range of clinical characteristics of patients, sources of isolates (specimens), nature of bacterial isolates and effect sizes. Patients with presumed or confirmed urinary tract infections took larger proportion of participants and midstream urine sample was the major source of bacterial isolates [20–29]. The rest sources of isolates were blood from septicemia and febrile patients [30–34], stool from patients with acute diarrhea [35–39], external ocular discharges from patients with ocular infections [40–42], vaginal discharges from pregnant women with infections [43–45], ear discharges with bacterial otitis media [46–48], and wound swabs from infected wounds [49, 50], among others. Some samples were taken from more than one source in a given patient [51–55]. Six of the included studies were retrospective analyses of secondary data [23, 28, 46, 47, 49, 52]. Majority of the isolates from stool were enteric gram-negative micro-organisms (salmonella and shigella) and gram positive enterococci. The average quality scores of studies ranged from 6 to 10 as per the Newcastle-Ottawa scale (Table 1).

**Table 1:** Characteristics of studies describing the resistance profile of clinical relevant bacterial isolates against ciprofloxacin

Study ID	Quality score	Year (pub)	Study Area	Study Design	Population (Clinical features)	Source of sample	Bacterial Category	Type of isolates	Number of isolates	No of resistant	(%)
Abamecha et al. [35]	8.5	2015	JUSH	CS	Hospitalized patients	Stool	Gram + Ve	*E. faecalis*	114	57	50.00
Abera et al. [51]	9	2016	FHRH	CS	In/outpatients with infections	Urine and Blood	Gram -Ve	*E. coli*	122	49	40.16
								*K. Pneumoniae*	49	28	57.14
								*P. mirabilis*	29	10	34.48
Alemseged et al. [43]	8	2015	ARH and MHC, Mekele	CS	Pregnant women	Vaginal swabs	Gram + Ve	GBS	19	1	5.26
Ali et al. [78]	8.5	2016	Gambella hospital	CS	STI suspected patients	Urethral or endo-cervical swabs	Gram -Ve	*N. gonorrhoeae*	21	6	28.57
Ameya et al. [36]	8	2018	Arba Minch province	CS	Under five children (diarrhea)	Stool	Gram -Ve	Salmonela	21	0	0.00
								Shigella	8	0	0.00
Denboba et al. [46]	7.5	2016	DRHRL	CS (R)	Patients with Otitis media	Ear discharges	Gram + Ve	*S. auerus*	102	1	0.98
							Gram -Ve	Pseudomonas spp	134	13	9.70
								Proteus spp	114	8	7.02
								*K. pneumoniae*	9	0	0.00
								*E. coli*	69	6	8.70
Assefa et al. [55]	8	2015	UoGH	CS	Dacryocystitis patients	Nasolacrim al discharge	Gram + Ve	*S. auerus*	6	0	0.00
								CoNS	9	4	44.44
Ayelign et al. [20]	8.5	2018	UoGH	CS	Pediatric patients with UTI	Urine specimens	Gram + Ve	*S. auerus*	8	0	0.00
							Gram -Ve	*E. coli*	45	8	17.78
								Pseudomonas spp	8	1	12.50
Bekele et al. [21]	7	2015	JUSH	CS	Catheterized patients	Urine samples (Catheter)	Gram -Ve	Pseudomonas spp	36	0	0.00
Bitew et al. [22]	8.5	2017	Arsho AML, AA	CS	Patients with UTI	Urine	Gram + Ve	*S. auerus*	9	3	33.33
								*E. faecalis*	14	1	7.14
							Gram -Ve	*E. coli*	135	68	50.37
								*K. pneumoniae*	18	3	16.67
Deribe et al. [23]	6.5	2017	Bahir Dar Regional HRLC	CS (R)	Patient with presumptive UTI	Urine	Gram + Ve	*S. auerus*	9	3	33.33
							Gram -Ve	*E. coli*	64	41	64.06
								*K. pneumoniae*	19	4	21.05
								Pseudomonas spp	8	0	0.00
								Proteus spp	6	4	66.67
Dereje et al. [24]	8.5	2017	Hamlin fistula hospital, AA	CS	Fistula patients (UTI)	Urine	Gram -Ve	*E. coli*	65	37	56.92
								*K. pneumoniae*	14	11	78.57
								Proteus spp	31	14	45.16
Derese et al. [25]	9.5	2016	DRH	CS	Pregnant women with UTI	Urine	Gram + Ve	CoNS	5	1	20.00
							Gram -Ve	*E. coli*	9	1	11.11
Dessie et al. [50]	9	2016	Selected referral hospitals, AA	CS	Surgical site infected patients	Wound swabs	Gram + Ve	*S. auerus*	19	3	15.79
							Gram -Ve	*E. coli*	24	16	66.67
								*K. pneumoniae*	10	2	20.00
								Pseudomonas spp	6	2	33.33
Eshetie et al. [26]	9.5	2015	UoGH	CS	Patients with UTI	Urine	Gram -Ve	*E. coli*	104	1	0.96
								*K. pneumoniae*	28	3	10.71
Gebrekidan et al. [37]	7.5	2015	Mekele hospital	CS	Outpatients with acute diarrhea	Stool	Gram -Ve	Shigella	15	1	6.67
Teweldemedihin [40]	8	2017	Quiha Ophthalmic Hospital	CS	Patients with ocular infections	Ocular specimens	Gram + Ve	*S. auerus*	40	5	12.50
								CoNS	31	3	9.68
								*E. faecalis*	8	1	12.50
								*K. Pneumonia*	7	1	14.29
								Pseudomonas spp	21	4	19.05
								*E. coli*	15	1	6.67
Getahun et al. [41]	10	2017	UoGH	CS	Patients with ocular infections	Ocular samples/ external	Gram + Ve	*S. auerus*	96	7	7.29
								CoNS	64	7	10.94
							Gram -2015Ve	*E. coli*	6	1	16.67
								*K. pneumoniae*	9	2	22.22
Gezmu et al. [27]	6	2016	Arba Minch Hospital	CS	Patients with UTI	Urine	Gram + Ve	*S. auerus*	10	3	30.00
							Gram -Ve	*E. coli*	20	4	20.00
								*K. pneumoniae*	8	2	25.00
Hailu et al. [34]	8	2016	Bahir Dar Reg HRLC	CS	Febrile patients	Blood	Gram + Ve	*S. auerus*	50	10	20.00
								CoNS	35	3	8.57
							Gram -Ve	*E. coli*	19	5	26.32
								*K. pneumoniae*	35	10	28.57
								Pseudomonas spp	15	2	13.33
Hailu et al. [49]	7.5	2016	Bahir Dar Regional HRLC	CS (R)	Patiets with infected wounds	wound swab	Gram + Ve	*S. auerus*	67	5	7.46
								*S. pyogens*	20	1	5.00
							Gram -Ve	*E. coli*	33	15	45.45
								*K. Pneumonia*	20	4	20.00
								Pseudomonas spp	26	5	19.23
								Proteus spp	22	5	22.73
Hailu et al. [47]	7.5	2016	Bahir Dar Regional HRLC	CS (R)	Patients with ear infections	Ear discharges	Gram + Ve	*S. auerus*	78	0	0.00
								CoNS	34	0	0.00
								*S .pneumonia*	7	0	0.00
							Gram -Ve	*E. coli*	7	1	14.29
								*K. Pneumoniae*	10	1	10.00
								Pseudomonas spp	88	7	7.95
								Proteus spp	65	3	4.62
Kumalo et al. [30]	7	2016	JUSH	CS	Sepsis patients	Blood	Gram + Ve	*S. auerus*	6	1	16.67
Lamboro et al. [38]	8.5	2016	JUSH	CS	Outpatients with diarrhea	Stool	Gram -Ve	Salmonella	19	0	0.00
Mengist et al. [44]	7	2016	JUSH	CS	Pregnant women	Anorectal and Vaginal	Gram + Ve	GBS	31	3	9.68
Mitku [28]	6.5	2017	DRHRL	CS (R)	Outpatients with UTI	Urine	Gram -Ve	*E. coli*	25	2	8.00
								*K. Pneumoniae*	7	1	14.29
								Proteus spp	6	1	16.67
Mulu et al. [52]	8.5	2017	DMRH	CS (R)	Any patients with infection	Non specific/ all types	Gram + Ve	*S. auerus*	13	6	46.15
							Gram -Ve	*E. coli*	22	4	18.18
								Pseudomonas spp	17	6	35.29
								Salmonella	16	4	25.00
								*N. gonorrheae*	8	13	61.53
Negussie et al. [31]	6.5	2015	Selected hospitals, AA	CS	Septicemia suspected children	Blood	Gram + Ve	*S. auerus*	13	4	30.77
								CoNS	11	2	18.18
							Gram -Ve	*K. pneumoniae*	9	4	44.44
Nigussie and Amsalu [29]	7.5	201	HURH	CS	Diabetic patients	Urine	Gram + Ve	*S. auerus*	6	3	50.00
								CoNS	8	4	50.00
							Gram -Ve	*E. coli*	11	2	18.18
Regassa et al. [53]	8	2015	JUSH	CS	CAP paients	Sputum and Blood	Gram + Ve	*S. auerus*	16	5	31.25
							Gram -Ve	Pseudomonas spp	10	2	20.00
								*K. pneumoniae*	8	0	0.00
Sahile et al. [54]	6	2016	JUSH	CS	Patients with surgical and gynecologic wound	Urine and wound swab	Gram + Ve	*S. auerus*	22	13	59.09
								CoNS	21	16	76.19
							Gram -Ve	*E. coli*	9	4	44.44
								Pseudomonas spp	8	4	50.00
								Proteus spp	7	3	42.86
Shiferaw et al. [42]	8.5	2015	BoruMeda Hospital	CS	Patients with ex-ocular infections	External ocular specimens	Gram + Ve	*S. auerus*	21	2	9.52
								CoNS	51	4	7.84
								*S. pneumoniae*	10	2	20.00
								*S. pyogens*	6	0	0.00
Terfasa and Jida [39]	8	2018	Nekemte referral hospital	CS	Diarrheal patients	Stool	Gram -Ve	Salmonella	30	2	6.67
								Shigella	9	0	0.00
Wasihun et al. [32]	8	2015	Mekelle hospital	CS	Febrile patients	Blood	Gram + Ve	*S. auerus*	54	21	38.89
								CoNS	44	11	25.00
							Gram -Ve	*E. coli*	16	1	6.25
								Salmonela	8	4	50.00
Wasihun et al. [33]	8.5	2015	Mekelle hospial	CS	Febrile patients	Blood	Gram + Ve	*S. auerus*	41	18	43.90
								CoNS	39	10	25.64
								*S. pyogens*	6	1	16.67
							Gram -Ve	*E. coli*	12	1	8.33
								Salmonella	8	1	12.50
Wasihun and Zemene [48]	8	2015	ARH	CS	Patients with otitis media	Ear discharges	Gram + Ve	*S. auerus*	46	10	21.74
								CoNS	17	9	52.94
								*S. pneumonia*	15	3	20.00
								*S. pyogens*	16	3	18.75
							Gram -Ve	Proteus spp	39	0	0.00
								Pseudomonas spp	27	10	37.04
								*K. pneumoniae*	18	2	11.11
								*E. coli*	6	1	16.67
Mulu et al. [45]	7	2015	FHRH	CS	Women with vaginal infections	Vaginal swabs	Gram + Ve	*S. auerus*	15	3	20.00
							Gram -Ve	*E. coli*	6	2	33.33
								Pseudomonas spp	7	0	0.00

*Abbreviations*: *CoNS* coagulase negative Staphylococci, *CS* cross-sectional, *R* retrospective, *HURH* Hawassa University Referral Hospital, *UoGH* University of Gondar Hospital; *JUSH* Jimma University Specialized Hospital, *DRHRL* Dessie Regional Health Research laboratory, *STI* sexually transmitted diseases, *UTI* Urinary tract infections, *ARH* Ayder Referral Hospital, *GBS* Group B Streptococci, *FHRH* Felege Hiwot Referral Hospital, *DMRH* Debre Markos Referral Hospital, *CAP Community Acquired Pneumonia*

### Study outcome measures

#### Gram-positive bacteria

**Fig. 2:**
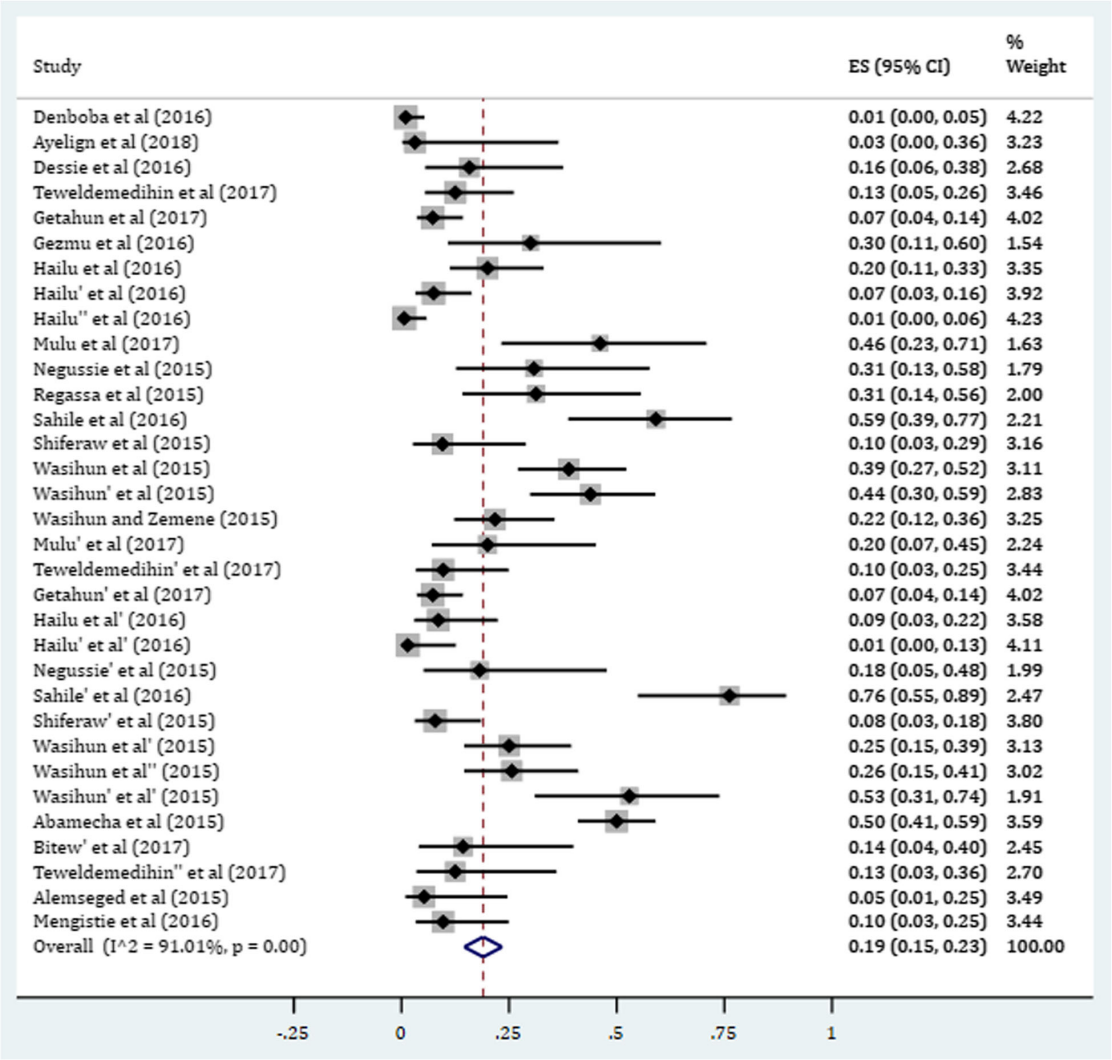
Pooled estimate of resistance in gram-positive bacteria against ciprofloxacin in Ethiopia

The overall estimate of resistance in selected gram-positive bacterial isolates against ciprofloxacin was found to be 19% (95% CI: 15, 23) (Fig. 2). In this category, the pooled estimates of resistance in *S. auerus* was 18.6% (95% CI: 13.5, 23.7) with degree of heterogeneity (I^2^), 88.18%. The resistance level of CoNS isolates was found to be 21.6% (95% CI: 12.4, 30.8). Higher degree of resistance was observed among *Enterococcus faecalis* with prevalence rate of 23.9%. There was low level of ciprofloxacin resistance in GBS isolates (7.40%) (Table 2).

**Table 2:** Subgroup analyses of resistance profiles of gram-positive and gram-negative bacterial isolates against ciprofloxacin

Category	Bacterial isolates	Pooled estimate (95% CI)
Gram positive bacteria	*S. aureus*	18.6% (13.5, 23.7)
	CoNS	21.6% (12.4, 30.8)
	*E. faecalis*	23.9% (7.9, 55.7)
	GBS	7.4% (0.2, 14.6)
Gram negative bacteria	*E. coli*	24.3% (14.2, 34.3)
	*K. pneumoniae*	23.2% (13.7, 32.7)
	*N. gonorrhea*	48.1% (18.3,87.9)
	Pseudomonas spp	14.1% (8.3, 19.8)
	Proteus spp	16.0% (7.9, 24.1)
	Other enteric pathogens (Shigella and salmonella)	6.3% (1.50, 11.1)

*GBS* Group B Streptococci, *CoNS* Coagulase Negative Staphylococci

#### Gram-negative bacteria

**Fig. 3:**
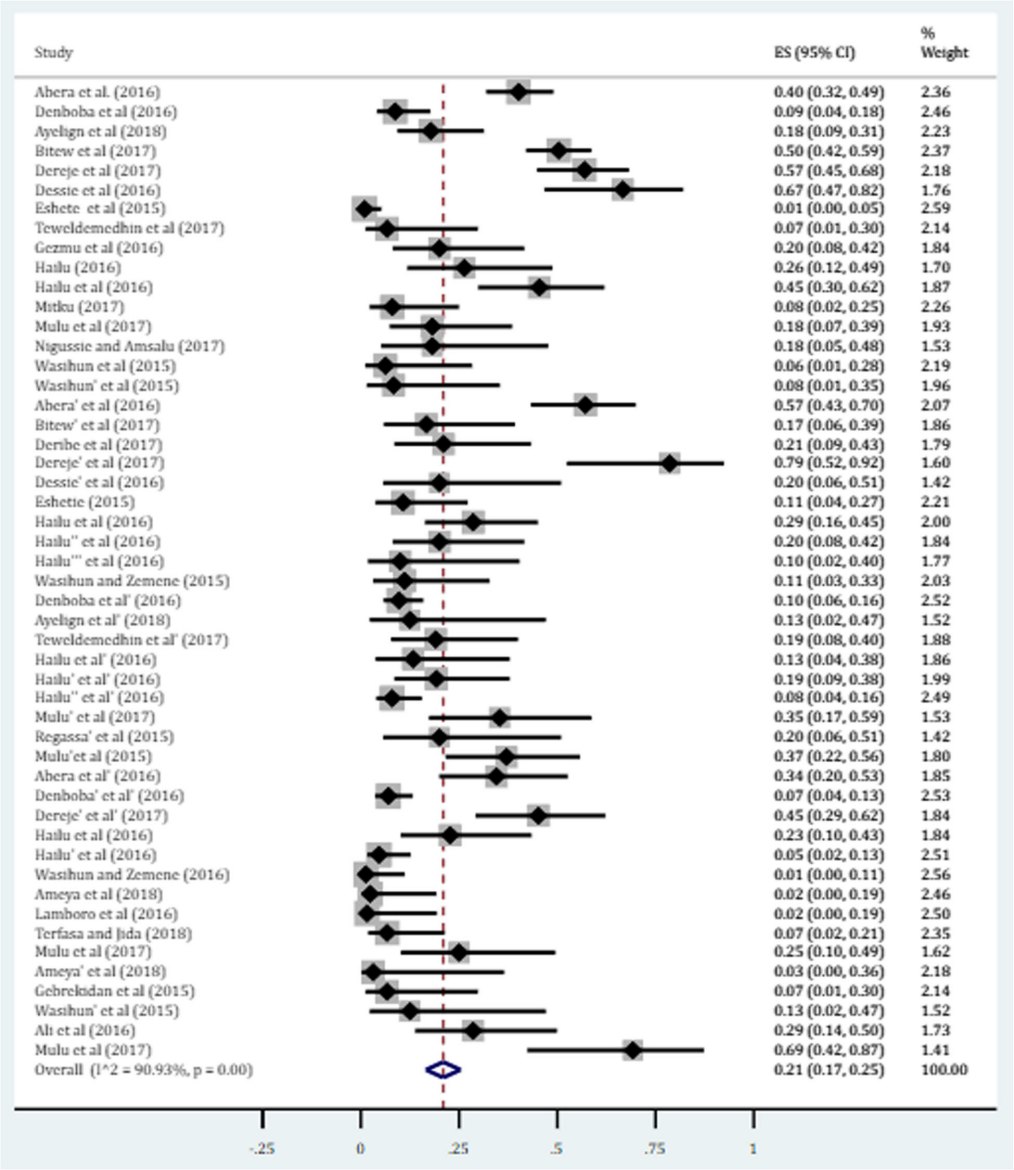
Forest plot depicting the resistance profile of gram-negative bacteria against ciprofloxacin

The gram-negative bacteria were the most common isolates obtained from several sources. The pooled estimate of resistance was 21% (95% CI: 17, 25) (Fig. 3). Among the selected isolates, higher degree of resistance was observed in *N. gonorrhea*, *E. coli* and *K. pneumoniae* with prevalence of 48.1, 24.3 and 23.2%, respectively. Besides, the pooled estimates of resistance in Proteus species (mainly *P. mirabilis*) and Pseudomonas species (primarily *P. aueroginosa*) against ciprofloxacin were found to be 16.0% (95% CI: 7.9, 24.1) and 14.1% (95% CI: 8.3, 19.8), respectively. The lowest degree of resistance was found among other gram negative enteric pathogens (salmonella and shigella) obtained from stool in patients with acute diarrhea. The overall estimate of resistance in these enteric species was found to be 6.3% (95% CI: 1.5, 11.1). Individual isolate (subgroup analysis) indicated that the prevalence of resistance in salmonella and shigella species was 8.1 and 5.8%, respectively (Table 2). In addition, we performed a univariate meta-regression model to identify whether sample size of individual isolates is a possible sources of heterogeneity; however, only the sampling distribution of *S. aureus* was found to be statistically significant (*p* value = 0.005) (Table 3).

**Table 3:** Univariate meta-regression model describing whether sample size is considered as a possible source of heterogeneity

Nature of bacterial isolates	Regression coefficients (95% CI)	*p* value
*S. aureus*	−0.003 (−0.005, −0.001)	0.005^*^
CoNS	−0.003 (−0.007, 0.002)	0.238
*E. coli*	0.001 (−0.001, 0.003)	0.200
Pseudomonas spp	0.000 (−0.001, 0.001)	0.577
*K. pneumoniae*	0.007 (0.000, 0.014)	0.059
Proteus spp	−0.003 (− 0.006, 0.000)	0.077
Other pathogens	−0.002 (− 0.008, 0.003)	0.450

^*^ Statistically significant at *p* value < 0.05

#### Source based subgroup and sensitivity analyses

There was a significant change on the degree of heterogeneity when we had excluded the expected outliers and studies with few numbers of isolates (less than five) per bacterium from the analyses. Very few sample size significantly affected the confidence intervals and point estimates. Therefore, we were subjected to exclude some of the studies for the meta-analysis at the initial scenario. We also conducted a subgroup analysis based on the source of bacterial isolates. These analyses further clarify whether there is a clinically significant difference in the degree of resistance of bacterial isolates across sources of specimens. Highest prevalence of resistant isolates was obtained from urine sample for CoNS (36%), *K. pneumoniae* (32%) and Proteus species (40%). Among the common sources, blood sample was endowed with larger proportion of resistant isolates of *S. aureus* 33% (95% CI: 20, 45). The resistance rates of *E. coli* and *Pseudomonas spp* from wound swabs and vaginal discharges, respectively, was found to be high (Table 4).

**Table 4:** Subgroup analysis of resistance profiles by the source of specimens

Common bacterial isolates, Proportion (95% CI)						
Common source	*S. aureus*	*CoNS*	*E.coli*	*Pseudomonas spp*	*Klebsiella spp*	*Proteus spp*
Urine	0.26 (0.03, 0.50)	0.36 (0.12, 0.84)	0.27 (0.10, 0.43)	0.02 (0.00, 0.05)	0.32 (0.12, 0.51)	0.40 (0.27, 0.52)
Blood	0.33 (0.20, 0.45)	0.19 (0.09, 0.28)	0.11(0.01, 0.22)	0.16 (0.02, 0.29)	0.23 (0.03, 0.43)	–
Ear discharges	0.03 (0.00,0.07)	0.26 (0.14, 0.76)	0.09 (0.03, 0.15)	0.09 (0.05, 0.13)	0.08 (0.00, 0.17)	0.04 (0.00, 0.08)
Wound swabs	0.08 (0.01,0.14)	–	0.56 (0.35, 0.76)	0.19 (0.04, 0.34)	0.20 (0.05, 0.34)	0.23 (0.05, 0.40)
Ocular discharges	0.09 (0.04, 0.13)	0.08 (0.04, 0.12)	0.08 (0.03, 0.20)	0.19 (0.02, 0.35)	0.18 (0.00, 0.36)	–
Vaginal discharges	0.20 (0.00, 0.40)	–	0.33 (0.00, 0.71)	0.37 (0.18, 0.55)	–	–

*CoNS* Coagulase negative staphylococci

#### Publication bias

**Fig. 4:**
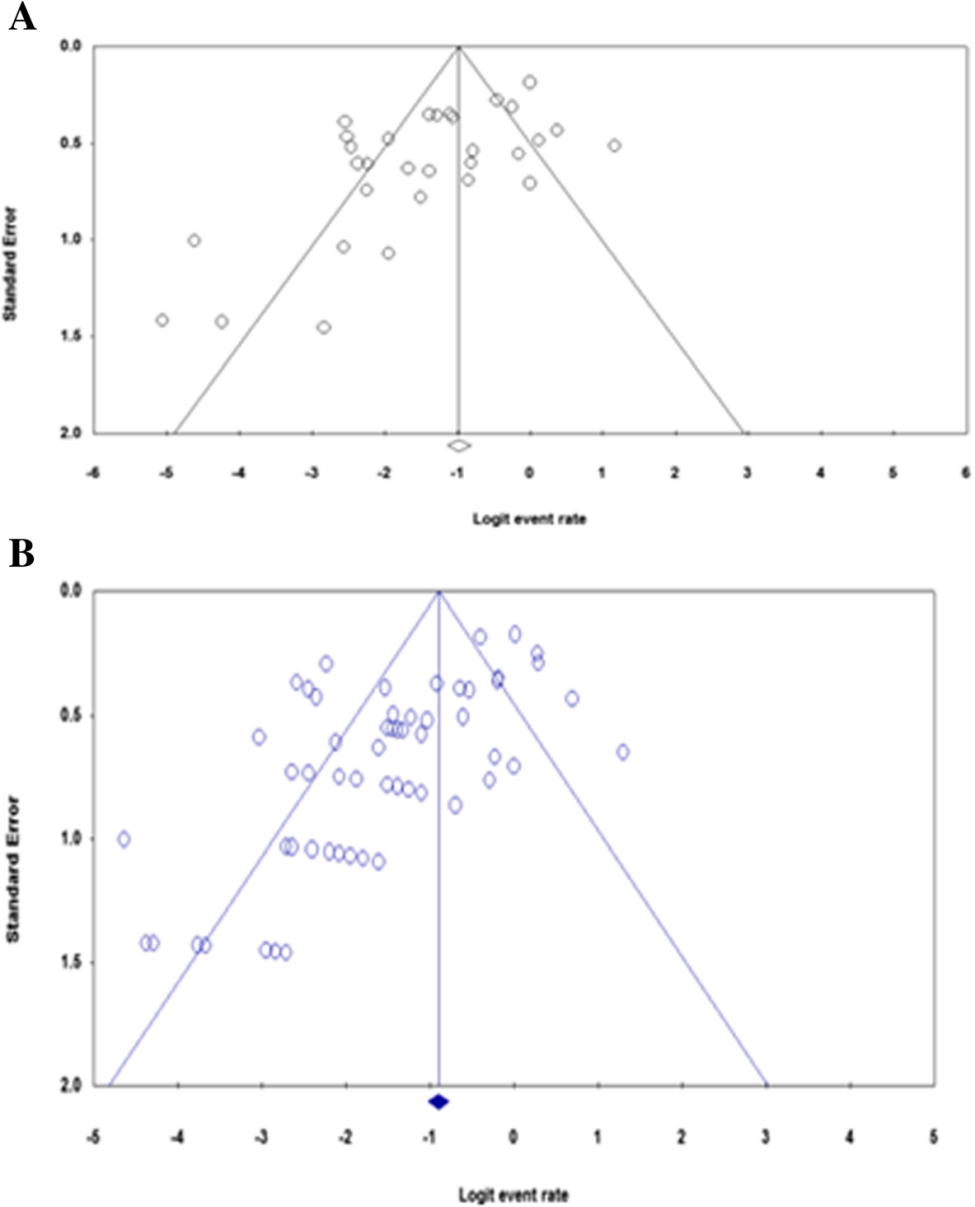
Funnel plot depicting publication bias **a** Studies describing gram-positive bacteria **b** Studies with gram-negative bacteria

Funnel plots of standard error with Logit event rate (proportion of resistant isolates) supplemented by statistical tests confirmed that there is some evidence of publication bias on studies reporting the prevalence of ciprofloxacin resistance among gram-positive (Begg’s test, *p* = 0.086; Egger’s test, *p* = 0.026) and gram- negative bacteria (Begg’s test, *p* = 0.06; Egger’s test, *p* = 0.0003) (Fig. 4a and b).

## Discussion

This systematic review and meta-analysis included 37 original studies addressing the prevalence of ciprofloxacin-resistant clinical isolates in Ethiopia within the specified timeframe. Regardless of the source and identity of isolates, the study revealed that one in five clinical isolates were found to be ciprofloxacin resistant in both gram-positive and gram-negative bacteria. *E. faecalis* from gram-positive bacteria and *N. gonorrhoea*, *E. coli* and *K. pneumoniae* from gram-negative bacteria exhibited higher prevalence of resistance as the meta-analysis indicated. The resistance estimate in other enteric pathogens (shigella and salmonella), obtained from stool samples, were found to be relatively less in Ethiopia. Urine and blood samples have been the major source of resistant isolates. In spite of relatively low level of resistance (8.1%) in Ethiopia, the emergence of ciprofloxacin resistance in common salmonella serotypes worldwide is becoming a serious public health concern. Besides, resistance to the first generation quinolones (nalidixic acid) has been associated with reduced efficacy of 6-fluorinated-quinolones such as ciprofloxacin [56, 57].

Routine antimicrobial surveillance data indicated the presence of strong relationship between antimicrobial use and resistance at a national level in Europe [58]. Even if quinolones are less likely to select for resistance compared to other natural antibiotics, highly level of use with some degree of misuse facilitates resistance selection and spread of quinolones resistance (QNR) genes to areas where the prevalence of resistance is found to be low [59–64]. Population mobility is a main factor in the spread of antimicrobial–resistant organisms [64]. To this end, Vernet et al. reported that 65% of *E. coli* strains isolated from patients who had traveled to India were found resistant to quinolones including ciprofloxacin [65]. Besides, surveillance data showed that resistance in *E. coli* and *K. pneumonia* has become consistently higher for antimicrobial agents that have been in use for long time in human and veterinary medicine [12]. In trajectory with our findings, significant increment in resistant level of *K. pneumonia* strain against ciprofloxacin was observed from 1998 to 2010 in United States [66]. Even if fluoroquinolones such as ciprofloxacin and ofloxacin have been highly effective in treating gonorrhea, the widespread and often inappropriate use leads to the emergence of fluoroquinolone resistant *N. gonorrhoea* [4, 67]. World Health Organization updated the current treatment profiles of *N. gonorrhea* as there has been an established resistance reports from various regions [67, 68].

To date, several mechanisms of quinolone resistance have been determined: modification of bacterial targets (DNA gyrase or topoisomerase IV) to which quinolones bind, decreased intracellular (bacterial) concentration due to an over-expression of active efflux pumps and enzymatic inactivation (acetylation) of quinolones, among others. Recently, mobile genetic elements carrying the QNR gene, which confer resistance to quinolones, have also been described [1, 69–71]. Amino acid changes in critical regions of the enzyme-DNA complex (quinolone resistance–determining region [QRDR]) reduce quinolone affinity for both targets [59–61]. QRDR mutation was identified in Enterococcus isolates; with serine being changed in gyrA83, gyrA87 and parC80. This result showed that gyrA and parC mutations could be important factors for high-level of resistance to such species against ciprofloxacin [70]. QNR proteins protect target enzymes from quinolone inhibition. The AAC(6′)-Ib-cr determinant acetylates several fluoroquinolones, such as norfloxacin and ciprofloxacin [69].

Plasmid-mediated quinolone resistance has been shown to compromise the bactericidal activity of fluoroquinolones when expressed in Enterobacteriaceae [72]. For example, plasmidic transfer of genes has resulted in spread of resistant strains among *E. coli*, *K. pneumoniae*, and Proteus species [73]. Jacoby et al. described the presence of QNR gene up on analyzing a long series of gram-negative microorganisms (mainly *K. pneumonia* and *E. coli*) from different geographical origins (19 countries) around the world) [62]. The development of fluoroquinolone resistance in staphylococci, *P. aeruginosa*, and other pathogens can also occur through alterations in DNA topoisomerase [74]. Besides, an endogenous system which actively transports quinolones out of the bacteria was described initially in *E. coli* and later in other gram-negative and gram-positive bacteria such as *S. aureus* [75, 76]. The QepA and OqxAB efflux pumps extrude fluoroquinolones from the bacterial cell [69]. Generally, the above-mentioned mechanisms of resistance have been established upon routine exposure of quinolones for treatment of many bacterial infections. AMR has resulted in increased morbidity, mortality, as well as direct and indirect healthcare costs in developing countries [14]. A notable example is an epidemic of infection associated with ciprofloxacin resistant *S. typhi* observed in Tajikistan [77].

## Conclusion

The study revealed that one in five gram-positive or gram-negative bacterial isolates developed resistance against ciprofloxacin in Ethiopia. Among gram-positive bacteria, high level of resistance was observed in Enterococci and CoNS whereas and relatively low degree of resistance was observed among GBS isolates. Within gram-negative bacteria, nearly half of isolates of *N. gonorrhoeae* was found ciprofloxacin resistant. From enterobacteriaceae isolates, *K. pneumonia* and *E. coli* showed relatively higher degree of ciprofloxacin resistance while shigella and salmonella had low level of resistance. Urine and Blood samples were the major sources of ciprofloxacin resistant isolates. Considering resistance estimates in to account, antimicrobial stewardship programs should be established in Ethiopian healthcare settings thereby preserves antimicrobials and contains AMR.

## Additional files

Additional file 1: **Table S1.** Completed PRISMA checklist. The checklist highlights the important components addressed while conducting systematic review and meta-analysis from observational studies. (DOC 65 kb)

Additional file 2: **Table S2.** Data abstraction format. The table presented the ways of data collection (study characteristics and outcome measures) in Microsoft excel format. It also contained a raw data for outcome analyses. (XLSX 27 kb)

## Abbreviations

AMR: Antimicrobial resistance
CoNS: Coagulase negative Staphylococci
GBS: Group B Streptococci
MeSH: Medical subject headings
PRISMA: Preferred Reporting Items for Systematic Review and Meta-analysis
QNR: Quinolone resistance
QRDR: Quinolone resistance determining region
WHO: World Health Organization
